# The impact of breast cancer biological subtyping on tumor size assessment by ultrasound and mammography - a retrospective multicenter cohort study of 6543 primary breast cancer patients

**DOI:** 10.1186/s12885-016-2426-7

**Published:** 2016-07-13

**Authors:** Roland Gregor Stein, Daniel Wollschläger, Rolf Kreienberg, Wolfgang Janni, Manfred Wischnewsky, Joachim Diessner, Tanja Stüber, Catharina Bartmann, Mathias Krockenberger, Jörg Wischhusen, Achim Wöckel, Maria Blettner, Lukas Schwentner, the BRENDA Study Group

**Affiliations:** Department of Obstetrics and Gynecology, Würzburg University Hospital, Josef-Schneider-Str. 4, 97080 Würzburg, Germany; Insititute of Medical Biostatistics, Epidemiology and Informatics (IMBEI), Mainz University Hospital, Mainz, Germany; Department of Obstetrics and Gynecology, Ulm University Hospital, Ulm, Germany; e-Science, Bremen University, Bremen, Germany

**Keywords:** Breast cancer, Ultrasound, Mammography, Tumor size, Histopathology

## Abstract

**Background:**

Mammography and ultrasound are the gold standard imaging techniques for preoperative assessment and for monitoring the efficacy of neoadjuvant chemotherapy in breast cancer. Maximum accuracy in predicting pathological tumor size non-invasively is critical for individualized therapy and surgical planning. We therefore aimed to assess the accuracy of tumor size measurement by ultrasound and mammography in a multicentered health services research study.

**Methods:**

We retrospectively analyzed data from 6543 patients with unifocal, unilateral primary breast cancer. The maximum tumor diameter was measured by ultrasound and/or mammographic imaging. All measurements were compared to final tumor diameter determined by postoperative histopathological examination. We compared the precision of each imaging method across different patient subgroups as well as the method-specific accuracy in each patient subgroup.

**Results:**

Overall, the correlation with histology was 0.61 for mammography and 0.60 for ultrasound. Both correlations were higher in pT2 cancers than in pT1 and pT3. Ultrasound as well as mammography revealed a significantly higher correlation with histology in invasive ductal compared to lobular cancers (*p* < 0.01). For invasive lobular cancers, the mammography showed better correlation with histology than ultrasound (*p* = 0.01), whereas there was no such advantage for invasive ductal cancers. Ultrasound was significantly superior for HR negative cancers (*p* < 0.001). HER2/neu positive cancers were also more precisely assessed by ultrasound (*p* < 0.001). The size of HER2/neu negative cancers could be more accurately predicted by mammography (*p* < 0.001).

**Conclusion:**

This multicentered health services research approach demonstrates that predicting tumor size by mammography and ultrasound provides accurate results. Biological tumor features do, however, affect the diagnostic precision.

## Background

Breast cancer remains the most common malignancy among women with an incidence of about 70,000 cases per year in Germany (http://www.krebsgesellschaft.de/basis-informationen-krebs/krebsarten/brustkrebs.html). Distinct biological subgroups of breast cancer show significantly different tumor growth and prognosis as well as therapeutic options [1]. The invasive carcinoma of no special type (NST), also known as invasive ductal carcinoma or ductal carcinoma NOS (not otherwise specified), accounts for about 70–80 % of breast cancers. Less common are invasive lobular cancers with 10–15 % of all breast cancers and rare subtypes such as medullary, tubular or mucinous carcinoma [2]. Using cDNA microarray analysis, Perou et al. defined different biological subgroups of breast cancers with impact on tumor biology and clinical appearance [1]: Luminal A and B breast cancers as well as HER2/neu positive and basal like breast cancer. Gene expression profiling is not yet part of routine tumor analysis But hormone receptor expression, HER2/neu overexpression and proliferation markers represent surrogate markers for biological breast cancer subgroups.

Tumor resection is still essential for therapy concepts in breast cancer care. In many cases, breast-conserving surgery can be performed instead of mastectomy. Incomplete or marginal tumor resection requires a re-resection. Imaging technologies are thus essential not only for diagnosis but also for preoperative assessment of breast cancer. Especially for non-palpable tumors, imaging plays an outstanding role.

Previous studies showed that mammography slightly overestimates tumor size, whereas ultrasound tends to underestimate tumor size [3]. Other groups found ultrasound to provide the more exact estimates for tumor size [4]. In these studies, there was no separate evaluation for the different biological subgroups of breast cancer. A single-center retrospective study of 121 patients [5] found that ultrasound-based assessments tend to underestimate in particular the size of invasive ductal cancer with ductal carcinoma in situ and invasive lobular as well as invasive ductal cancers. Bosch et al. published a prospective study that found ultrasound to be the best predictor of histological tumor size compared to mammography and physical examination. As ultrasound underestimated the tumor size, they suggested a formula for calculating the probable histological tumor size: Sonographic tumor size (mm) +3 mm [6]. Ultrasound seems to be especially good in the assessment of tumors with less than 30 mm diameter [7]. Ramirez and colleagues found good correlations between ultrasound, mammography and especially MRI with histological tumor size [8].

According to German guidelines for breast cancer diagnostics and treatment, mammography is the standard imaging tool [9]. In case of high breast density (ACR 3–4), an ultrasound examination should be added to achieve higher sensitivity [10]. Both mammography and ultrasound are standard diagnostic tools for breast cancer assessment [11, 12]. The role of magnetic resonance imaging (MRI) of the breast as preoperative assessment is controversial: In a metaanalysis of 9 clinical studies, Houssami and colleagues found that MRI did not reduce re-excisions but significantly increased the rate of modified radical mastectomies (MRM) [13, 14]. They suggest that a routine MRI in breast cancer patients could do more harm than good [13]. Though preoperative bilateral breast MRI could reduce the risk of a contralateral cancer recurrence, Yi et al. could not find any difference in local-regional recurrence rates [15].

The role of MRI in breast cancer imaging is still controversial while ultrasound and mammography remain the gold standard in care. We therefore aimed to investigate accuracy of the gold standard imaging techniques in a multicenter health services research approach investigating breast cancer imaging in a large daily routine cohort of patients.

## Methods

We retrospectively analyzed data from 6543 breast cancer patients who were part of the BRENDA I study population. Patients with unifocal, unilateral primary breast cancer were included in the BRENDA I study. Data were collected from 1992 until 2008 at Ulm University Hospital and from 2002 until 2008 in 16 associated German breast cancer centers certified by the German Cancer Society. The study protocol was approved by the Ethics committee of the University of Ulm. Patients gave informed consent. Data regarding maximum tumor diameter in preoperative ultrasound, mammography, as well as histological tumor diameter were collected.

Patients were excluded from the analysis if they received neoadjuvant chemotherapy, if they suffered from bilateral, multicenter or inflammatory breast cancer as well as non-invasive tumors. In case of missing diagnostic data, the patients were also excluded.

The maximum tumor diameter was measured by imaging as well as by pathologic examination. In case of follow-up resections, tumor diameters were added excluding ductal carcinoma in situ (DCIS) and lobular carcinoma in situ (LCIS).

Endocrine responsiveness was categorized according to the 2007 St. Gallen Consensus Criteria [16].

### Statistical analysis

Statistical analysis was performed using R (version 3.1 [17]). Patient characteristics were described with percentages, mean values and standard deviations. Precision (variability) and accuracy (systematic bias) of imaging methods were analyzed separately. Precision of mammography and ultrasound tumor size measurements were assessed by calculating Pearson’s correlation coefficient with histological tumor size. T-tests were used to compare the independent correlation coefficients of the same imaging method between patient groups. To compare the correlation coefficients between imaging methods for the same patient group, Williams’ test for the difference between two dependent correlations sharing one variable (histological tumor size) was used. Accuracy of imaging methods was assessed by their respective mean differences to histology measurements. Numerical results were complemented by visual evaluation of Bland-Altman plots that show the difference between the tumor diameter as measured by two methods against the mean of both measurements.

To provide a detailed evaluation of precision of tumor size measurements by mammography as well as ultrasound with respect to histology, we performed several types of comparisons: A) Comparisons of each imaging method across different patient groups. B) Comparison between mammography and ultrasound within one patient group. Patient groups were defined by either their age, or by different tumor characteristics like histological sub-type. We finally compared the precision of the detection of a 20 mm tumor diameter cutoff (C.). The impact of patient age on imaging was analyzed, respectively (D.).

## Results

### Description of the study population

Six thousand five hundred forty-three patients were eligible for the study. The mean age at diagnosis was 61.9 (SD 13.0 years). Three thousand eight hundred fifty-nine patients were stage pT1 , 2469 with pT2 and 217 patients with pT3. Four thousand two hundred ten patients (64.3 %) showed pN0 status. 10.8 % of the tumors were graded as G1, the majority of tumors were G2 (61.8 %) and 27.3 % were G3 carcinomas. 14.4 % of the tumors were hormone receptor (HR) negative. 14.8 % of the tumors overexpressed HER2.

#### Comparisons of each imaging method across different patient groups

##### Mean difference between *sonographic* and histological tumor size

**Fig. 1:**
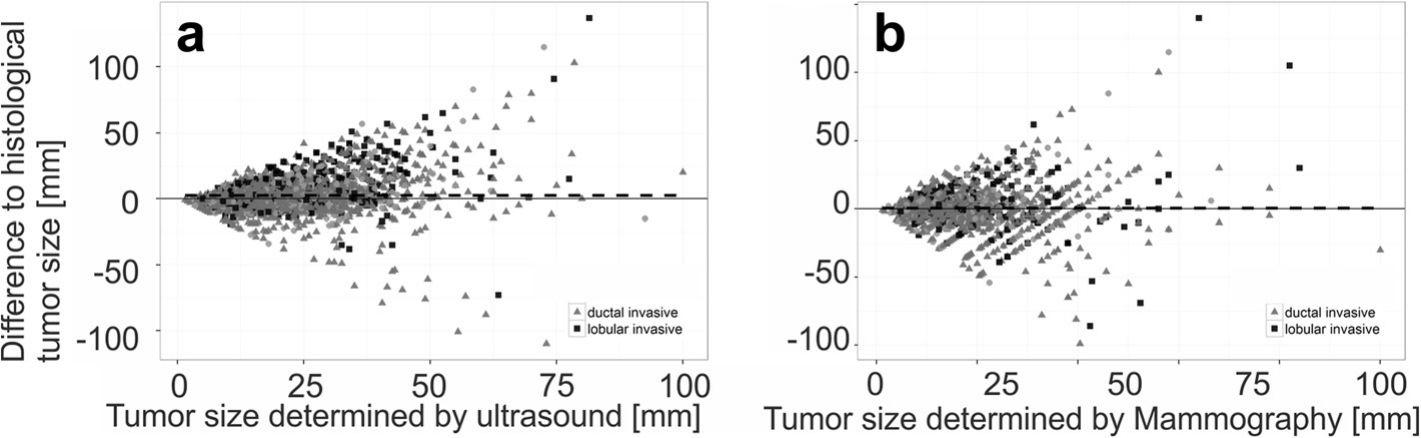
Difference between sonographic, mammographic tumor size. Bland-Altman Diagrams of the Differences between tumor size as measured by ultrasound (**a**) and mammography (**b**) plotted against their respective mean value. Histological subtypes are indicated

The distributions of measured tumor size were generally unimodal and slightly right-skewed. The mean tumor diameter determined by ultrasound was 18.3 mm (SD 9.6 mm), whereas the histological mean tumor diameter was 20.8 mm (SD 12.3 mm). Data are summarized in Table 1. A Bland-Altman plot (Fig. 1a) indicates that measurement differences were proportional to tumor size with invasive lobular tumors being over-represented among tumors that are underestimated by ultrasound: Among 198 tumors underestimated by more than 20 mm, 68 (34 %) were invasive lobular cancers. Among 62 tumors overestimated by ultrasound by more than 20 mm, only 4 (6 %) were invasive lobular cancers. Among 5642 tumors neither over- nor underestimated by more than 20 mm, 665 (12 %) were invasive lobular cancers (*p* < 0.001).

**Table 1:** Comparison of ultrasound and histology

	Mean histologic diameter	Mean sonographic diameter	Mean difference	Mean relative difference (% sonogaphic tumor diameter)	SD Histologic diameter	SD Sonographic diameter	*N*
Overall	20.78	18.25	2.534	21.08	12.29	9.629	5902
Endocrine non-responsive	23.77	21.10	2.666	17.24	13.98	10.205	848
Incomplete endocrine responsive	19.88	18.18	1.702	16.91	11.60	10.408	1224
Highly endocine responsive	20.43	17.62	2.811	23.44	12.00	9.078	3817
Not applicable	14.69	23.46	−8.769	−29.05	12.85	16.179	13
Ductal invasive	20.41	18.31	2.097	18.28	11.69	9.663	4257
Lobular invasive	23.42	17.72	5.699	43.13	14.97	9.627	737
G1	15.03	14.48	0.5521	11.15	9.824	9.191	652
G2	20.08	17.61	2.4675	21.97	11.678	9.317	3636
G3	24.69	21.21	3.4854	23.08	13.289	9.697	1614
pT1	13.52	14.03	−0.5067	6.024	5.159	7.066	3450
pT2	28.45	23.34	5.1141	35.658	6.989	8.347	2265
pT3	61.84	34.45	27.3904	122.220	17.761	16.055	187
Age <50 years	20.06	17.70	2.353	21.99	12.40	9.127	1295
Age 50–70 years	19.65	17.12	2.509	21.80	11.91	9.477	3042
Age >70 years	23.00	10.50	12.500	93.33	21.21	6.364	2

Overall, ultrasound underestimated the histological tumor size with a mean difference of 2.5 mm. This result also appeared in HR positive and HR negative tumors as well as in invasive ductal and invasive lobular cancers. There was a tendency towards decreasing sonographic accuracy in G3 high grade cancers.

Ultrasound accuracy was strongly dependent on tumor size: In pT1 cancers, the sonographic tumor diameter was higher than the histological tumor diameter. pT2 and pT3 cancers always had larger histological tumor diameters than determined by ultrasound.

##### Mean difference between *mammographic* and histological tumor size

The overall mean histological diameter for patients examined by mammography was 21.0 mm, and the mean mammographic diameter was 20.4 mm. An overview of the mammography data is shown in Table 2. A Bland-Altman plot (Fig. 1b) indicates that measurement differences were proportional to tumor size with invasive lobular tumors being over-represented among tumors that are underestimated by mammography: Among 110 tumors underestimated by more than 20 mm, 28 (25 %) were invasive lobular cancers. Among 110 tumors overestimated by mammography by more than 20 mm, only 12 (11 %) were invasive lobular tumors. Among 4010 tumors neither over- nor underestimated by more than 20 mm, 434 (11 %) were invasive lobular cancers (*p* < 0.001).

**Table 2:** Comparison of mammography and histology

	Mean histologic diameter	Mean mammographic diameter	Mean difference	Mean relative difference (% mammographic tumor diameter)	SD Histologic diameter	SD mammographic diameter	*N*
Overall	21.01	20.41	0.6012	14.85	12.5	11.91	4230
Endocrine non-responsive	23.33	23.42	−0.09015	11.08	13.603	13.431	599
Incomplete endocrine responsive	20.45	20.05	0.39892	13.50	12.347	11.308	930
Highly endocine responsive	20.70	19.87	0.82993	16.15	12.246	11.663	2693
Not applicable	17.50	18.62	−1.125	16.52	7.426	7.425	8
Ductal invasive	20.59	20.30	0.2864	12.99	11.63	11.55	3132
Lobular invasive	23.61	20.68	2.9325	31.22	15.97	13.32	474
G1	15.00	15.44	−0.435	7.551	10.66	10.22	423
G2	20.16	19.65	0.5078	15.023	11.44	11.32	2623
G3	25.05	23.87	1.1782	17.078	14.02	12.78	1184
pT1	13.52	15.13	−1.612	3.881	5.242	8.134	2408
pT2	28.51	26.11	2.399	24.427	6.995	10.127	1693
pT3	62.6	44.28	18.318	93.960	21.265	23.583	129
Age <50 years	21.11	20.05	1.0606	16.54	12.54	11.53	808
Age 50–70 years	19.65	19.10	0.5579	16.12	12.18	11.49	2210
Age >70 years	23.44	23.06	0.3757	11.44	12.67	12.47	1211

In both invasive ductal and invasive lobular cancer size was overall underestimated by mammography.

For mammography, tumor size was an important factor for the observed accuracy. pT1 cancers with a mean histologic diameter of 13.5 mm were overestimated in mammography while the opposite was true for pT2 and pT3. The difference peaked in the pT3 group with a mean histologic diameter of 62.6 mm and a mean difference of 18.3 mm. Similarly, G1 cancers with a mean histological tumor diameter of 15.0 mm appeared larger in mammography whereas the size of G2 and G3 cancers was underestimated. Again, the peak mean difference was found in G3 cancers.

#### Comparison between mammography and ultrasound within one patient group

The correlation coefficients between histology, ultrasound and mammography for the respective subgroups are shown in Table 3.

**Table 3:** Correlation of tumor diameter in histology, Ultrasound and mammography

Correlation overall	Correlation coefficient	*N*
Histology – Ultrasound	0.5981	5902
Histology – Mammography	0.605	4230
Ultrasound – Mammography	0.7369	3757
Correlation in ductal invasive cancer		
Histology – Ultrasound	0.6045	4257
Histology – Mammography	0.6157	3132
Ultrasound – Mammography	0.7319	2784
Correlation in lobular invasive cancer		
Histology – Ultrasound	0.5209	737
Histology – Mammography	0.5271	474
Ultrasound – Mammography	0.7012	424
Correlation for pT1		
Histology – Ultrasound	0.3178	3450
Histology – Mammography	0.2528	2408
Ultrasound – Mammography	0.5922	2121
Correlation for pT2		
Histology – Ultrasound	0.3938	2265
Histology – Mammography	0.3864	1693
Ultrasound – Mammography	0.6349	1534
Correlation for pT3		
Histology – Ultrasound	0.1344	187
Histology – Mammography	0.1989	129
Ultrasound – Mammography	0.5527	102
Correlation for endocrine non-responsive cancer (HR negative)		
Histology – Ultrasound	0.6427	848
Histology – Mammography	0.5252	599
Ultrasound – Mammography	0.7436	538
Correlation for incomplete endocrine responsive cancer (HR positive)		
Histology – Ultrasound	0.5212	1224
Histology – Mammography	0.5781	930
Ultrasound – Mammography	0.7092	811
Correlation for highly endocrine responsive cancer (HR positive)		
Histology – Ultrasound	0.6124	3817
Histology – Mammography	0.6334	2693
Ultrasound – Mammography	0.7407	2401
Correlation for HER2/neu positive cancer		
Histology – Ultrasound	0.6345	797
Histology – Mammography	0.4375	565
Ultrasound – Mammography	0.7408	507
Correlation for HER2/neu negative cancer		
Histology – Ultrasound	0.5958	4587
Histology – Mammography	0.6466	3278
Ultrasound – Mammography	0 0.7332	2956
Correlation for patients aged <50 years		
Histology - Ultrasound	0.4658	1295
Histology - Mammography	0.5158	808
Ultrasound - Mammography	0.6922	729
Correlation for patients aged 50–70 years		
Histology - Ultrasound	0.5970	3042
Histology - Mammography	0.5809	2210
Ultrasound - Mammography	0.7250	1941
Correlation for patients aged >70 years		
Histology - Ultrasound	0.6700	1563
Histology - Mammography	0.6752	1211
Ultrasound - Mammography	0.7583	1086

Correlations with histology are shown for pairwise data, whereas correlations between Ultrasound and mammography required complete datasets

As we sought to evaluate the precision of different diagnostic methods in breast cancer subgroups, we compared the correlations of ultrasound with histology, of mammography with histology and, respectively, of ultrasound with mammography.

Overall, the analyses comparing histology and ultrasound or histology and mammography showed no significant differences between the two non-invasive techniques (*p* = 0.18).

Both, ultrasound and mammography showed significantly higher correlations with histology in invasive ductal compared to invasive lobular cancers (*p* = 0.002, 3.07/*p* = 0.008).

Ultrasound and histology further showed a significantly better correlation for pT2 compared to pT1 cancers (*p* = 0.001). This correlation was also highly significantly superior for pT2 compared to pT3 cancers (*p* = 0.0002). Equivalent results could be detected in the correlation of mammography and histology, which was also significantly higher for pT2 compared to pT1 (*p* < 0.001) or compared to pT3 (*p* = 0.026).

In the subgroup of invasive lobular cancers, histology showed a significantly higher correlation with mammography than with ultrasound (*p* = 0.01). There was no such difference in the invasive ductal cancer subgroup.

For HR negative cancers, ultrasound showed a significantly higher correlation with histology (*p* < 0.001). Size estimates by mammography were, however, significantly more accurate for HR positive than for HR negative non-responsive cancers, as evidenced by the superior correlation with histology (*p* = 0.0003).

Still, in both HR negative and HR positive cancers, mammography was inferior to ultrasound regarding the correlation with histology (*p* < 0.001/*p* < 0.001 ).

The correlation of mammography with histology was, however, significantly better for the HER2/neu negative than for the HER2/neu positive subgroup (*p* < 0.001). For the HER2/neu negative subgroup, mammography data showed a significantly higher correlation with histology whereas ultrasound was less precise (*p* < 0.001). In the HER2/neu positive subgroup, however, ultrasound came significantly closer to the histological size determination (*p* = 0.0001).

*Ultrasound tends to underestimate the tumor size in invasive lobular cancers. Invasive lobular cancers showed a significantly higher percentage of grossly underestimated tumors (>35 mm difference to histology).*

#### Precision of ultrasound and mammography for 20 mm cutoff detection

For further therapy, 20 mm tumor size is an important cutoff. We thus analyzed the sensitivity of mammography and ultrasound in detecting this tumor size cutoff. For detection of tumor sizes over 20 mm, ultrasound was slightly more specific (0.752 versus 0.703) and slightly more sensitive than mammography (0.824 versus 0.799). Ultrasound showed a higher cutoff detection rate (0.225 versus 0.172), superior positive predictive (0.555 versus 0.424) values. Mammography was superior only at negative predictive values (0.919 versus 0.927).

#### Patient age impacts both ultrasound and mammography precision

The results in relation to patient age are shown in Table 4. As breast density decreases in older patients, we analyzed the results in different age groups. Patients aged <50 years, 50–70 years and >70 years were compared respectively.

**Table 4:** Patient age impacts both ultrasound and mammography precision

Age (years) versus T stadium	pT1	pT2	pT3
<50	0.63591	0.33570	0.02839
50-70	0.63337	0.33754	0.02908
>70	0.46590	0.48902	0.04509
Age (years) versus Grading	G1	G2	G3
<50	0.095	0.530	0.375
50-70	0.122	0.627	0.251
>70	0.092	0.674	0.234
Age (years) versus HR expression	HR negative	HR incompletely responsive	HR positive
<50	0.20	0.23	0.57
50-70	0.14	0.21	0.65
>70	0.10	0.21	0.69
Age (years) versus HER2/neu expression	HER2/neu negative	HER2/neu positive	
<50	0.82	0.18	
50-70	0.85	0.15	
>70	0.88	0.12	
Age (years) versus histological subtype	Ductal invasive	Lobular invasive	
<50	0.75	0.093	
50-70	0.72	0.133	
>70	0.71	0.128	
Age (years)	HER2/neu negative	HER2/neu positive	
<50	0.82	0.18	
50-70	0.85	0.15	
>70	0.88	0.12	

Relative Quantifications for T stadium, Grading, HR expression, HER2/neu expression or histological subtype in relation to age are shown

Higher patient age correlated with higher tumor size and respective T stage. Patients aged <50 years showed more HR negative cancers compared to older patients. The percentage of invasive ductal and lobular cancers was comparable in all age groups.

Both mammography and ultrasound were highly significantly superior for patients aged >70 years compared to patients aged 50–70 years (*p* < 0.01). Both mammography and sonography achieved the lowest precision in patients aged <50 years compared to patients aged 50–70 years (*p* = 0.024/*p* = <0.001).

Still, the histology correlation of mammography and ultrasound did not significantly differ in any age group.

## Discussion

In our study, the overall correlation between histology and mammography was 0.61 for mammography and 0.60 for ultrasound and thus did not show any significant difference in terms of precision of tumor diameter measurement (*p* = 0.18). Both ultrasound and mammography did show a significantly higher correlation with histological tumor diameter in invasive ductal compared to invasive lobular cancers (*p* = 0.002 / *p* = 0.008). For invasive lobular cancers, mammography turned out to be superior to ultrasound with respect to the correlation with histological tumor diameter (*p* = 0.01), whereas there was no advantage in the invasive ductal cancer subgroup. The analysis was focused on tumors detected by respective imaging. pT2 cancers could generally be assessed more precisely by both ultrasound and mammography whereas pT1 or pT3 showed more deviation. This result could be biased by the more accurate palpation of T2 tumors. While HR positive cancers did not show a difference between the precision of ultrasound and mammography, HR negative cancers show a highly significant advantage for ultrasound (*p* < 0.001). HER2/neu positive cancers also showed the superiority of ultrasound (*p* < 0.001) whereas mammography was superior in predicting the size of HER2/neu negative cancers (*p* < 0.001).

In line with Gruber et al. [5], we found ultrasound to underestimate histological tumor diameter. MRI data were not available for our study. Nevertheless, by comparing ultrasound and mammography data with histopathological findings, the precision of imaging-based tumor size determination could be assessed for the various biological subclasses of breast cancer. This showed that HR expression as well as HER2/neu overexpression impacts the precision achieved by imaging.

Hieken et al. [4] published that both ultrasound and mammography underestimated tumor size. In 180 cases of invasive breast cancers, they found ultrasound to be more accurate. In clear contrast to their results, we could show distinct differences of imaging precision in invasive ductal and invasive lobular cancers and thus provide evidence for the importance of biological cancer subgroups for imaging.

Dummin and colleagues [3] found, that ultrasound underestimates breast cancer size. Mammography turned out to be the most precise tool for predicting histological tumor size. However, they did not compare different biological cancer subgroups regarding the correlations between histological, sonographic and mammographic tumor diameter.

It has to be considered that our retrospective study is an analysis of longitudinal study data. Further studies should investigate not only the maximum tumor diameter but for example three-dimensional tumor size. Improved ultrasound technologies such as 3D ultrasound make this possible. Our analysis is based on a large set of patient data, even though ultrasound and mammography data were not available for all patients. Furthermore, there was no information about breast density in imaging according to the American college of radiology (ACR). A great advantage of the longitudinal BRENDA I study is that the data were collected under realistic daily routine conditions. Precise data also exist for exact histological tumor diameter and all histological subtypes of breast cancer are represented. We could thus show that both ultrasound and mammography are reasonably precise in assessing tumor size. Mammography seems favorable for HER2/neu negative and invasive lobular cancers. Ultrasound is more precise for HER2/neu positive and HR negative invasive ductal cancers.

## Conclusion

We provide evidence that the prediction of tumor size by ultrasound and mammography in breast cancer is reliable in this large multicentered daily routine cohort of primary breast cancer patients. Nevertheless, our data suggest that inherent features of individual tumor subgroups influence the non-invasive assessment of tumor size. Taking this into consideration may further improve the interpretation of imaging data for therapeutic decisions.

## Abbreviations

ACR: American College of Radiology; CLIS / LIN: carcinoma lobulare in situ; DCIS: ductal carcinoma in situ; Fig.: figure; HR: hormone receptor (Estrogen- and Progesterone-Receptor); MRI: magnetic resonance imaging; MRM: modified radical mastectomy; NOS: not otherwise specified breast cancer; NST: no special type breast cancer; SD: standard deviation
